# Education Research: Virtual Patient Management Conference for Epilepsy Surgery in the Post–COVID-19 Era: An Educational Perspective

**DOI:** 10.1212/NE9.0000000000200089

**Published:** 2023-09-25

**Authors:** Thomas Pecha, Sharonya Shetty, Abhishek Kengen, Jay Gavvala, Atul Maheshwari

**Affiliations:** From the Baylor College of Medicine (T.P., S.S., A.K., A.M.); and McGovern Medical School (J.G.), Houston, TX.

## Abstract

**Background and Objectives:**

The primary objective of this study was to assess the potential educational value of a virtual patient management conference (PMC) with the introduction of inclusive anonymous polling at a comprehensive epilepsy center. The secondary objective was to evaluate differences between faculty and trainee polling results.

**Methods:**

Two online surveys were sent 1 year apart seeking opinions about a transition to virtual PMC and completed by virtual PMC faculty participants (including representatives from neurology, neurosurgery, and neuropsychology). One online survey was sent to trainees (medical students, residents, and fellows) to assess the educational value of the conference. Anonymous electronic polls surveying treatment options were completed by both faculty and trainees after each virtual PMC presentation but before discussing the case. The results were collected and analyzed over 16 months, including over the course of 1 academic year. The degree of consensus was determined by the maximum percentage of votes that a single choice received.

**Results:**

Eleven faculty and 22 trainees responded to their respective surveys. The initial faculty survey revealed that 60.0% of faculty had an “excellent” or “very good” experience with virtual PMC; 1 year later, this proportion increased to 100.0% while trainees reported 90.9%. Each virtual PMC component, including perceived standard of care, was found to be “excellent” or “very good” by most faculty and trainees, and most (91% faculty, 63.7% trainees) were equally comfortable or more comfortable expressing opinions during the virtual discussion. During virtual PMC polls, faculty members were significantly more likely to vote for vagus nerve stimulation as a treatment option, while trainees were more likely to opt for responsive neurostimulation. Linear regression over the course of the academic year showed stable consensus over time for both faculty and trainees; however, the match between faculty and trainee consensus significantly increased over the academic year.

**Discussion:**

Our results demonstrate that the virtual PMC constitutes an effective educational experience as an alternative to in-person conferences for the management of patients with drug-resistant epilepsy.

## Introduction

The transition from in-person visits to telemedicine has been among the most notable changes to the structure of medical encounters precipitated by the coronavirus disease 2019 (COVID-19) pandemic, both in the educational and clinical space. Virtual encounters between and among clinicians and patients have been swiftly implemented across the medical landscape in recent years, and the effects of these transitions are beginning to be appreciated. One particular area of interest involves identifying the repercussions of virtual multidisciplinary approaches on trainee education and patient care. Multidisciplinary teams (MDTs) have long been an effective tool for managing complex patient care, especially in the realm of oncology, where they often take the form of multidisciplinary tumor boards (MTBs). Review by an MTB or similar multidisciplinary management strategy has been shown to result in a changed diagnosis in over 42% of patients with breast cancer and has been associated with significantly increased 5-year overall survival in patients with esophageal cancer.^1,2^ The quality and effectiveness of virtual multidisciplinary panels are now beginning to be assessed, and early results have been reassuring. A retrospective cohort study analyzing the outcomes of patients with heart failure discussed in a virtual multidisciplinary clinic during the COVID-19 pandemic found no significant increase in mortality or major cardiovascular events compared with in-person discussions.^3^

With the implementation of virtual multidisciplinary panels now increasing, the effect on trainee education can also be evaluated. In March 2020, a similar transition to a virtual format occurred with the multidisciplinary patient management conference (PMC) for the Baylor College of Medicine Comprehensive Epilepsy Center, where patients who are considered for epilepsy surgery are discussed among various faculty and trainees. Of the 5 major pillars that are generally used in adult education, including instrumental, humanistic, transformative, social, and motivational learning, the PMC environment is most conducive toward the application of social learning models.^4^ A trainee in social learning theory benefits from highlighting the “Zone of Proximal Development,” which is the distance between the learner's independent problem solving and their potential for development with guidance from a more capable peer or teacher.^5^ According to this theory, the learner develops through interaction with other people in a collaborative setting.^2^ Another benefit within the social learning model is the concept of “communities of practice (CoP),” which has 3 components: the common interest of the members of the group, the knowledge shared between them, and the interactions within the group that ultimately lead to learning and development.^5^

In this framework, the PMC is a CoP with faculty and trainees in attendance, facilitating interactions with the common goal of developing together through mutual learning. While multidisciplinary panels were not created to specifically meet this need, they create a space where trainees can interact with their peers as well as learn from discussions between faculty about complex cases. Furthermore, the virtual setting may facilitate participation from learners, rather than just from faculty. Creating this environment where learners feel comfortable sharing their opinions without fear of judgment can greatly add to the support learners receive from the group experience.

Here, we aim to describe the transition of an epilepsy PMC at a Level 4 comprehensive epilepsy center to a virtual platform and evaluate the degree to which a virtual PMC was perceived as educational by trainees, provided an appropriate standard of care, and allowed for greater involvement in the discussion by its participants. In addition to constituting a valuable resource for trainee education, virtual epilepsy PMCs may represent a viable, effective option for discussing individual patient management. Further refinement of this process will aid in expanding the capabilities and benefits of the PMC.

## Methods

Before the COVID-19 pandemic, PMCs at the Baylor Comprehensive Epilepsy Center were conducted in-person, with faculty and trainees convening from 8:15 to 9:15 am on Tuesday mornings every 1–2 weeks. Clinical cases would be presented, and any pertinent findings would be displayed and discussed by participants before a discussion commenced. The first virtual conference was performed on March 24, 2020, using a video conferencing platform (Zoom, Zoom Video Communications).

**Figure 1:**
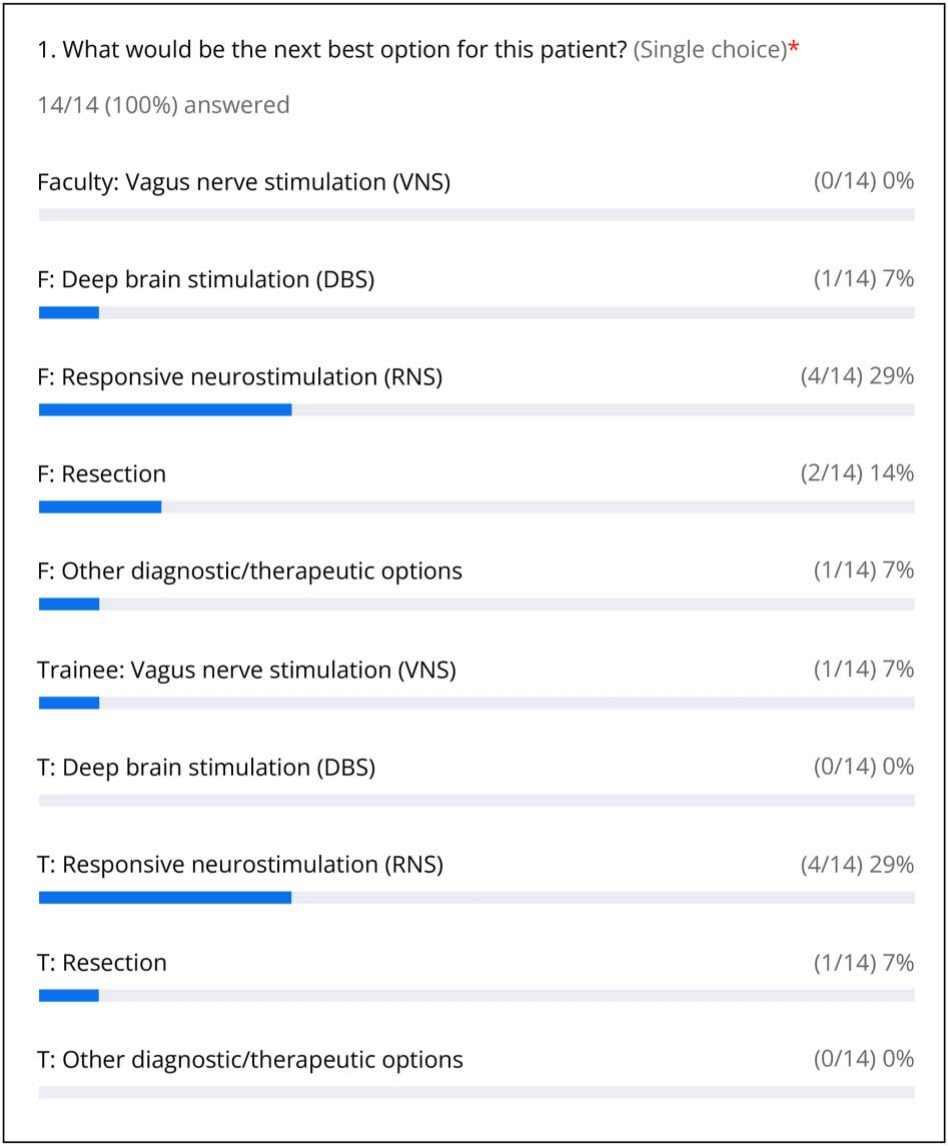
Sample Polling Results Representative PMC poll results, showing number and percentage of faculty and trainee votes for various management options. PMC = patient management conference.

After the shift to virtual PMCs, weekly meetings were held through Zoom, with a designated epileptologist (A.M.) functioning as moderator. The primary physician presenting the case would be the patient's outpatient epileptologist, with or without the involvement and assistance of a clinical neurophysiology or epilepsy fellow. Video-EEG findings of patients' seizures would be viewed simultaneously by faculty and trainees to study seizure semiology, as well as any relevant prior studies including but not limited to MRI, PET, SPECT, neuropsychological testing, Wada testing, and magnetoencephalography results. After a case was presented, faculty members (with representatives from neurosurgery, neurology, and neuropsychology) and trainees (including medical students, residents, and fellows) would vote on identical anonymous polls displayed by the moderator, consisting of 2–5 varying patient management options which were sent to the moderator before the PMC. The question was always “What would be the next best option for this patient? (Single Choice).” One of the choices typically included “other diagnostic/therapeutic options.” Of note, the Zoom platform limited the total number of options to 10, so there was a maximum of 5 options separately for faculty and trainees. After the results of the polls were made visible to participants (Figure 1), faculty and trainees would then discuss prospective management plans.

On January 8, 2021, over 9 months after the initial transition to virtual PMCs, a 5-question anonymous electronic survey was created and circulated to any faculty member who had participated in the virtual conference. Opinions were sought concerning faculty members' overall impressions of the virtual PMC, PMC duration, and polling system, as well as any suggestions for modification.

After 12 months of further virtual PMC use, a second anonymous electronic survey was sent to participating faculty members, again assessing opinions regarding overall experience with virtual PMCs, opinions on PMC length, and suggestions for improvement as well as how comfortable faculty felt sharing their opinions, their satisfaction with standard of care, and their rating of the quality of various PMC components (video recordings, review of EEGs and radiology, discussions, etc). A modified version of this survey was sent to trainees (medical students, residents, and fellows), which included an additional question about the educational value of virtual PMCs. Responses from all 3 surveys were gathered using Survey Monkey (surveymonkey.com) and subsequently underwent quantitative and qualitative analyses.

Faculty and trainee votes from the virtual PMC polls were also gathered and separately analyzed. For each poll, the number of votes for each patient management option was tallied; a χ^2^ test was used to determine which patient management options received significantly different proportions of votes from faculty and trainees. The degree of consensus was calculated by the maximum percentage of votes that a single choice received. The choice with the greatest consensus was defined as the option with the greatest percentage of votes, independently calculated for the faculty and the trainees. The probability of matching between faculty and trainee votes was calculated as a moving average over 5 cases, with 6 levels of matching probability (0%, 20%, 40%, 60%, 80%, and 100%). Linear regression was used to determine whether the degree of faculty or trainee consensus or probability of matching changed over the course of the academic year. This consensus comparison demonstrated to what extent trainees were able to learn from faculty responses and how closely their recommendations came to match those of experienced faculty over time.

A χ^2^ test was also used to determine which treatments were more likely to be chosen when faculty and trainee polls agreed. If multiple treatment options had an equally high vote count, each choice was counted separately. Finally, a Kruskal-Wallis test with a Dunn test for multiple comparisons was performed on faculty and trainee votes to evaluate the potential for order effect on voting.

### Data Availability

Data not provided in this article because of space limitations may be shared (anonymized) at the request of any qualified investigator for purposes of replicating procedures and results.

## Results

**Figure 2:**
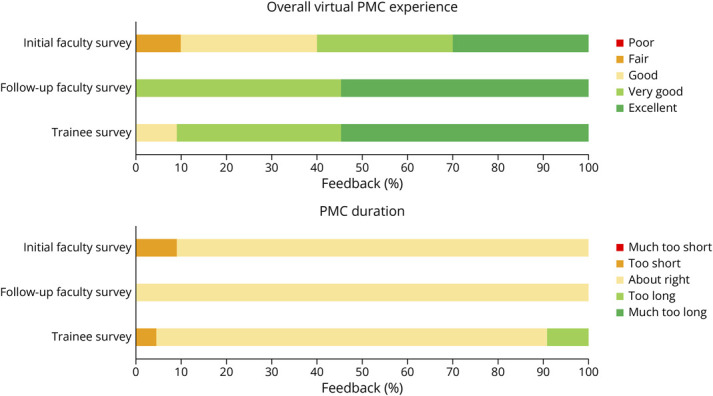
Percent of Faculty and Trainees Reporting Feedback for 2 Shared Survey Questions PMC = patient management conference.

The initial faculty survey in January 2021 yielded 11 responses and achieved a 100% response rate, with most of the faculty reporting positive overall experiences with the virtual PMC (90%) and its duration (91%, Figure 2, eTable 1, links.lww.com/NE9/A41). The faculty included a mix of 9 epileptologists (with 1–2 years of clinical neurophysiology and/or epilepsy fellowship training), 1 neurosurgeon, and 1 neuropsychologist. Comments for improvement centered on minimizing interruptions and the possibility of presenting postsurgical patient follow-ups. In response to this initial survey, several changes were initiated: (1) after sharing the poll results, a fellow or resident would be asked to first explain the rationale for his or her polling choice before opening up the discussion to the group; (2) polling was extended to all cases, not just the first case; (3) participation was further encouraged and streamlined by the use of the chat option and “raise hand” function in Zoom; and (4) polling responses were saved for further analysis. The potential for presenting postsurgical follow-up cases was discussed but ultimately declined because of lack of time.

**Figure 3:**
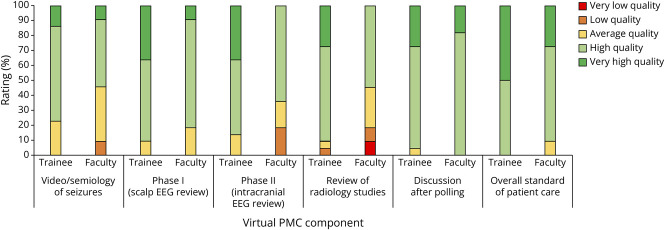
Percent of Faculty and Trainees Rating Various Virtual PMC Components by Quality No significant difference between faculty and trainees for any virtual PMC component. PMC = patient management conference.

One year after implementing these changes, a follow-up faculty survey and a new trainee survey were sent out on February 8, 2022. The follow-up faculty survey yielded 11 total responses (84.6% response rate). The overall positive experience with the virtual PMC increased to 100%, and 100% agreed that the PMC was the proper length of time (Figure 2, eTable 1, links.lww.com/NE9/A41). In total, 18.2% of faculty felt “much more comfortable” expressing their opinions during discussion compared with in-person PMCs, 36.4% felt “more comfortable,” 36.4% felt “equally comfortable,” and 9.1% (1 faculty member) had no prior in-person PMC experience (eTable 3). Furthermore, most faculty rated individual components of the virtual PMC as either “high” or “very high” quality (Figure 3, eTable 4). Comments by faculty described the virtual format as beneficial in reducing domination of the discussion by a few participants and again indicated a desire for postsurgical follow-ups to be presented for educational purposes.

The trainee survey yielded 22 responses from neurology, neurosurgery, and neuropsychology training programs as well as Baylor College of Medicine medical students. The response rate was 48.9% overall, with 8 of 12 fellows (66.7%), 11 of 30 residents (36.7%), and 3 of 6 (50.0%) medical students responding. All fellows (5 clinical neurophysiology, 5 epilepsy, 1 functional neurosurgery, and 1 neuropsychology) were invited to attend each PMC throughout the year, whereas residents would rotate 1–2 times per year for 1 month at a time, and medical students would only rotate once per year for 2 weeks at a time. All the trainees rated their overall experience with virtual PMCs to be positive to some degree, and most (86.4%) believed the PMCs to be of a proper duration (Figure 2, eTable 2, links.lww.com/NE9/A41). Compared with faculty, only 4.6% of trainees felt “much more comfortable” expressing their opinions during the discussion compared to in-person PMCs, 27.3% felt “more comfortable,” 31.8% felt “equally comfortable,” 4.6% felt “less comfortable,” and the remainder had no prior in-person experience (eTable 3). Like faculty, most trainees agreed each PMC component was either “high” or “very high” quality, including perceived overall standard of care (Figure 3, eTable 4). Finally, 40.9% of trainees found virtual PMCs to be an “extremely valuable” educational experience, 45.5% found it “very valuable,” and 13.6% found it “somewhat valuable” for their educational experience. Anonymous trainee comments focused on adding more time for discussion, including a postconference session for case-related instructional material, and encouraging greater trainee participation.

Data compiled from the patient management polls from 12 months of virtual PMCs showed several differences in management strategy preferences. An average number of 1.52 ± 0.08 (mean ± SEM, range 1–4) cases per PMC were presented and discussed at each conference. Faculty (n = 624, with 8.0 ± 0.2 votes per case) were significantly more likely to recommend vagus nerve stimulation (VNS) than trainees (*p* = 0.039), while trainees (n = 440, with 5.4 ± 0.2 votes per case) were significantly more likely to recommend responsive neurostimulation (RNS) (*p* = 0.012, Table 1). There were no statistically significant differences between voting preferences for DBS, nonspecific neuromodulation, intracranial evaluations, or surgical resection (Table 1). In cases in which faculty and trainee polls had consensus on the same management option, Phase 2 intracranial evaluation was significantly more likely to be agreed upon (*p* = 0.01), while other options, including RNS (*p* = 0.06) and VNS (*p* = 0.5), were not (χ^2^ test).

**Table 1:**
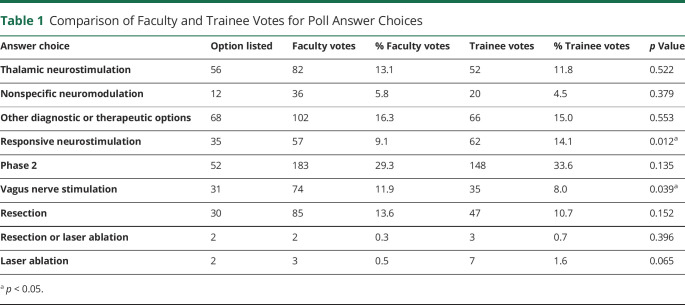
Comparison of Faculty and Trainee Votes for Poll Answer Choices

Answer choice	Option listed	Faculty votes	% Faculty votes	Trainee votes	% Trainee votes	*p* Value
Thalamic neurostimulation	56	82	13.1	52	11.8	0.522
Nonspecific neuromodulation	12	36	5.8	20	4.5	0.379
Other diagnostic or therapeutic options	68	102	16.3	66	15.0	0.553
Responsive neurostimulation	35	57	9.1	62	14.1	0.012^a^
Phase 2	52	183	29.3	148	33.6	0.135
Vagus nerve stimulation	31	74	11.9	35	8.0	0.039^a^
Resection	30	85	13.6	47	10.7	0.152
Resection or laser ablation	2	2	0.3	3	0.7	0.396
Laser ablation	2	3	0.5	7	1.6	0.065

a *p* < 0.05.

**Figure 4:**
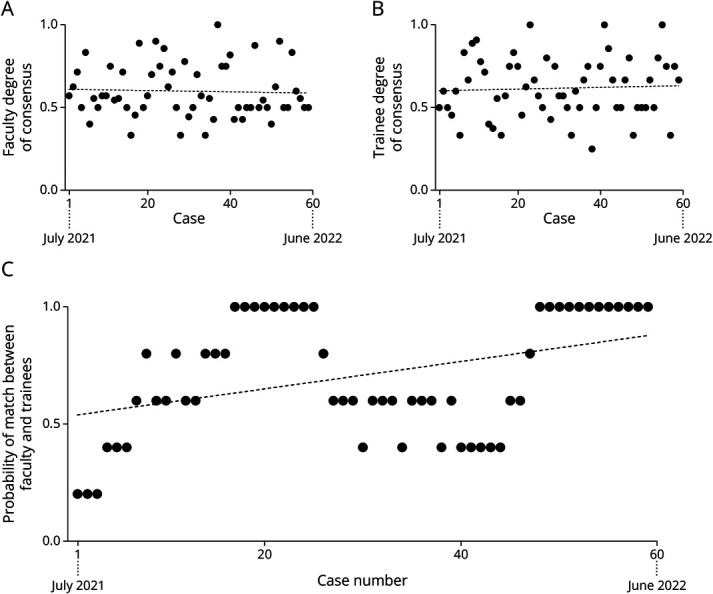
Degree of Consensus (Within and Between Faculty and Trainees) for Poll Votes No change in faculty (A) or trainee (B) consensus over time. (C) Significantly increased probability of matching consensus choices between faculty and trainees over the academic year (*r*^2^ = 0.15, *p* = 0.002).

We next asked whether the degree of consensus changed within and between faculty and trainees using linear regression analysis based on the choice with the maximum percentage of votes (a higher percentage infers higher consensus). Over the course of the academic year (between July 2021 and June 2022), the degree of consensus did not significantly change over time within both faculty and trainee groups (Figure 4, A and B). However, the overall probability of the choice with the highest degree of consensus matching between faculty and trainees significantly increased over the course of the academic year (*r*^2^ = 0.15, *p* = 0.002), with a bimodal peak seen in the middle of the year and at the end of the year (Figure 4C).

**Figure 5:**
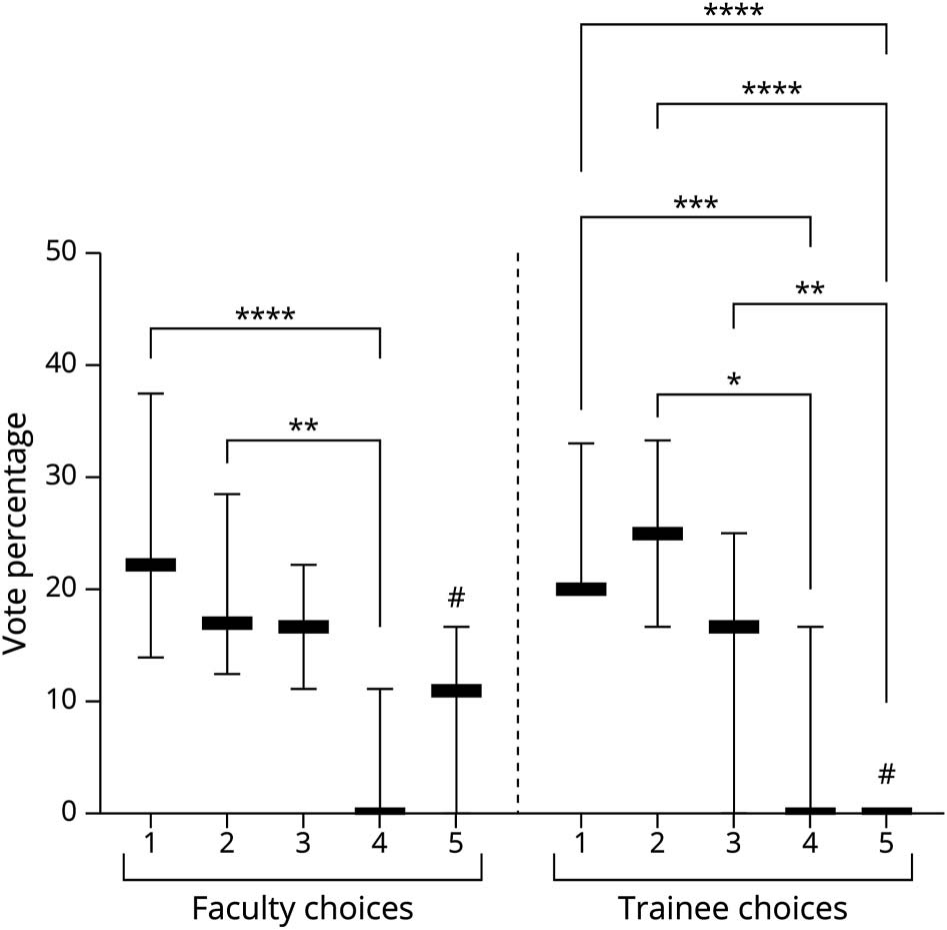
Percentage of Votes That Each Answer Choice Received Between Faculty and Trainees Faculty more frequently chose Choice 5 (most commonly “other diagnostic/therapeutic options”) compared with trainees (#, median 11.1% vs 0%, *p* = 0.004, Kruskal-Wallis with Dunn multiple comparison test). Within faculty choices, there was less of a significant order effect (2 significant preferences for options 1 and 2 over option 4) compared with trainees (5 significant preferences for options 1–3 over 4–5). *p < 0.05, **p < 0.01, ***p < 0.001, ****p < 0.0001.

Finally, evaluating for a potential order effect, there was a statistically significant difference in the percentage of votes between choices 1 and 2 and choice 4 among faculty votes (*p* < 0.05, Kruskal-Wallis test with Dunn post hoc test for multiple comparisons). For trainees, there were significant differences between choices 1 vs 4 (*p* < 0.0001) and 2 vs 4 (*p* = 0.02) as well as between 1 vs 5 (*p* < 0.0001), 2 vs 5 (*p* < 0.0001), and 3 vs 5 (*p* = 0.005, Figure 5). In addition, faculty were more likely to choose option 5 (typically “other diagnostic/therapeutic options”) than trainees (*p* = 0.004).

## Discussion

In this study, we aimed to investigate how transitioning to a virtual epilepsy PMC affected the faculty/trainee experience and trainee education. We found that over 85% of trainees found the virtual PMC to be either very valuable or extremely valuable for their education. Most faculty and trainees had very positive overall experiences, felt equally or more comfortable participating in the discussion compared with in-person conferences, and found the virtual PMC components to be of generally high quality. The increase in overall PMC experience rating among faculty supports the reforms instituted after the first poll, but increased familiarity with the virtual system over time was also a potential contributing factor. The virtual nature of the PMC, particularly the anonymous polling system, also likely contributed to the greater reported comfort during discussion among faculty and trainees. Such a system allows votes to be cast anonymously which may allow participants to recommend options that are not in line with conventional or dominant opinions. Importantly, nearly every participant found the overall standard of patient care and discussion to be of high or very high quality. Altogether, these findings suggest that the transition to a virtual platform was perceived as maintaining an overall high standard of care while also providing a valuable educational experience for trainees.

The responses to our survey align with the general principles of social theories of learning, indicating the utility of this method of teaching in meeting the need for creating a social context for learning.^5^ Using the “Zone of Proximal Development” as a model, the polling system serves as a direct representation of the learner's skills at the time, while faculty's answers represent the guidance for the trainees. Furthermore, the implementation of asking one of the fellows or residents for their rationale in choosing a specific option created an environment where reflection was encouraged, further stimulating learning and development, as well as fostering an environment where the learners were being included as part of the larger community and team. In this way, the virtual PMC serves as a virtual CoP, an integral component of social learning theory.^5^

One problem that previous studies have identified is a barrier which participants feel when participating in an ongoing discussion.^6^ In our study, faculty and trainees were able to participate despite not actively speaking due to the implementation of polling. The added reflection of trainees further added to their learning and development. The virtual PMC served as a catalyst for these opportunities, which created an environment where learners felt more engaged and comfortable sharing their thoughts and views regarding ongoing discussions (eTable 3, links.lww.com/NE9/A41). These contributions facilitate a critical dimension for a CoP in social learning theory: interactions within the group that lead to learning and development.

Faculty and trainee perceptions of the virtual PMC align with previous studies, many of which have focused on similar virtual transitions in a range of specialties. A study of 50 physicians using virtual MDT found that over 80% believed that virtual MDTs provided equivalent standard of care and 60% would support its use after the end of the pandemic; in addition, participants reported virtual conferences to be equal to or better than in-person meetings in each conference.^7^ A separate analysis of virtual tumor boards found that clinicians were generally satisfied with the virtual system and believed it to be a valuable method for discussion of complex cases.^8^ Similarly, an Italian study reviewing the transition to virtual lung cancer-specific MTBs found that participants also reported virtual MTBs as providing equivalent standard of care.^9^ An Oxford musculoskeletal oncology MTB polled its members and found that 83% believed diagnostic decision-making to be unchanged, with over 75% of participants reporting satisfaction with the level of discussion.^10^ Our findings strongly support the notion that virtual epilepsy PMCs provide similarly high levels of satisfaction among participants while retaining excellent perceived standard of care and quality of discussion for both faculty and trainees. Although our study was not designed to evaluate superiority of virtual over in-person PMC, our results show that the virtual option represents a viable and practical alternative in which both faculty and trainees feel comfortable discussing, learning, and participating.

Our results also found that faculty members were significantly more likely to vote for VNS and less likely to vote for RNS compared with trainees. This may be due to the relative novelty of RNS, which might hold more appeal for trainees as a promising technology. By contrast, the longer history of VNS, its less invasive nature, and greater clinical experience might explain the proclivity for faculty to recommend it more than trainees.^11,12^ There are rapidly evolving data regarding neurostimulation and center-specific variation in preferences, so the degree of variability could also be related to trainees not having enough time to internalize center-specific norms of practice. Interestingly, in cases in which faculty and trainees agreed on the same treatment, phase 2 intracranial evaluation was significantly more likely to be the agreed-upon choice, suggesting strong consensus when there was a need to seek further information before initiating a definitive treatment.

The degree of consensus within both the faculty and trainees over time remained stable at around 60% on average throughout the academic year. However, the consensus between faculty and trainees over the course of the academic year had a weakly positive but statistically significant relationship (Figure 4). These findings suggest that the trainees are showing evidence of learning throughout the year, with their overall consensus aligning more closely with faculty consensus over time. There was considerable variability, with a notable dip in faculty-trainee consensus around early spring. This may be the evidence of greater independent thinking or simply related to normal variation based on a heterogeneous learner group. Specifically, the trainee group included fellows, residents, and medical students, but only the fellows have regular continuity and participation in PMC. Future studies with a greater sample size of trainees consisting solely of fellows may be able to show even greater evidence of learning over time.

In addition to these specific educational benefits, virtual PMCs also surmount several inherent challenges of in-person meetings. Most appreciably, virtual meetings preclude the need for participants to assemble in a single physical location, thereby potentially improving attendance and participation and allowing for more patient cases to be discussed.^13-15^ This, in turn, may reduce the delay to multidisciplinary discussion, improve data sharing, and optimize clinical trial referrals while maintaining high-quality decision-making.^9,16^ Virtual conferences, however, are not without drawbacks. These include the potential for decreased personal connections, nonverbal communication, and camaraderie because of the lack of in-person interaction as well as technical difficulties and issues with data confidentiality.^14,17^

This study was limited by its narrow and retrospective focus and was also not designed to determine whether the transition to virtual PMCs affected patient outcomes. However, our results clearly indicate that faculty and trainees overwhelmingly perceive standard of care to be excellent. Another limitation involved the structure of prediscussion polling: Patients' ultimate treatments were not exclusively dictated by the results of the polls; rather, the voting system served as a starting point for further debate and nonetheless formed an integral part of the patient management process. In addition, the 1-way analysis of variance results indicate that the poll options contributed by the presenting faculty contained inherent bias due to a potential predisposition to place more popular recommendations earlier in the answer choices. Trainees seemed to be particularly susceptible to this order effect, and future attempts to account for this could involve randomization of the voting options to avoid any response bias. Finally, the follow-up faculty survey contained several questions about virtual PMC component quality not present in the initial faculty survey, preventing a true comparison from being made concerning changes in opinions about virtual PMC quality over time.

Future work should evaluate how often a discussion leads to a different management plan than was dictated by polling, which was outside the scope of this study. In addition, some suggestions from the comments from faculty and trainee survey results could be implemented for continued quality improvement: improved radiologic review, addition of a postconference reflection session for focused trainee education, occasional presentation of postsurgical follow-up, and software adjustments to allow for more options. The implementation of regular follow-up surveys would further identify areas for improvement and monitor quality improvement progress.

In conclusion, our study suggests that the virtual PMC constitutes a promising, viable method for discussing patients with drug-resistant epilepsy. In addition to allowing for more open discussion among participants, virtual PMCs overcome many challenges of in-person settings while maintaining excellent perceived quality of care and creating a valuable educational experience for trainees.

## Appendix Authors

**Table:**

Name	Location	Contribution
Thomas Pecha, BS	Baylor College of Medicine, Houston, TX	Study concept or design; analysis or interpretation of data
Sharonya Shetty, BS	Baylor College of Medicine, Houston, TX	Study concept or design
Abhishek Kengen, MD	Baylor College of Medicine, Houston, TX	Analysis or interpretation of data
Jay Gavvala, MD	McGovern Medical School, Houston, TX	Major role in the acquisition of data; analysis or interpretation of data
Atul Maheshwari, MD	Baylor College of Medicine, Houston, TX	Major role in the acquisition of data; study concept or design; analysis or interpretation of data

## Glossary

CoP: community of practice
COVID-19: coronavirus disease 2019
MDT: multidisciplinary team
MTB: multidisciplinary tumor board
PMC: patient management conference
RNS: responsive neurostimulation
VNS: vagus nerve stimulation

## References

[1] Garcia D, Spruill LS, Irshad A, Wood J, Kepecs D, Klauber-DeMore N. The value of a second opinion for breast cancer patients referred to a national cancer institute (NCI)-designated cancer center with a multidisciplinary breast tumor board. Ann Surg Oncol. 2018;25(10):2953-2957. doi:10.1245/s10434-018-6599-y 29971672 PMC6132422

[2] Hsu PK, Chien LI, Huang CS, et al. Treatment patterns and outcomes in patients with esophageal cancer: an analysis of a multidisciplinary tumor board database. Ann Surg Oncol. 2022;29(1):572-585. doi:10.1245/s10434-021-10568-z 34387767

[3] Zhao M, Qin D, Cataldo G, et al. Virtual multidisciplinary care for heart failure patients with cardiac resynchronization therapy devices during the Coronavirus Disease 2019 pandemic. Int J Cardiol Heart Vasc. 2021;34:100811. doi:10.1016/j.ijcha.2021.100811 34095452 PMC8165087

[4] Taylor DCM, Hamdy H. Adult learning theories: implications for learning and teaching in medical education: AMEE Guide No. 83. Med Teach. 2013;35(11):e1561-e1572. doi:10.3109/0142159X.2013.828153 24004029

[5] Mukhalalati B, Elshami S, Eljaam M, Hussain FN, Bishawi AH. Applications of social theories of learning in health professions education programs: a scoping review. Front Med. 2022;9:912751. doi:10.3389/fmed.2022.912751 PMC9367215 35966845

[6] McLoughlin C, Patel KD, O'Callaghan T, Reeves S. The use of virtual communities of practice to improve interprofessional collaboration and education: findings from an integrated review. J Interprof Care. 2018;32(2):136-142. doi:10.1080/13561820.2017.1377692 29161155

[7] Sidpra J, Chhabda S, Gaier C, Alwis A, Kumar N, Mankad K. Virtual multidisciplinary team meetings in the age of COVID-19: an effective and pragmatic alternative. Quant Imaging Med Surg. 2020;10(6):1204-1207. doi:10.21037/qims-20-638 32550130 PMC7276359

[8] Shea CM, Teal R, Haynes-Maslow L, et al. Assessing the feasibility of a virtual tumor board program: a case study. J Healthc Manag Am Coll Healthc Exec. 2014;59(3):177-193. PMC4116610 24988672

[9] Gebbia V, Guarini A, Piazza D, et al. Virtual multidisciplinary tumor boards: a narrative review focused on lung cancer. Pulm Ther. 2021;7(2):295-308. doi:10.1007/s41030-021-00163-8 34089169 PMC8177259

[10] Rajasekaran RB, Whitwell D, Cosker TDA, Gibbons CLMH, Carr A. Will virtual multidisciplinary team meetings become the norm for musculoskeletal oncology care following the COVID-19 pandemic? Experience from a tertiary sarcoma centre. BMC Musculoskelet Disord. 2021;22(1):18. doi:10.1186/s12891-020-03925-8 33402136 PMC7784619

[11] Harrison M, Marra CA, Bansback N. Preferences for “new” treatments diminish in the face of ambiguity. Health Econ. 2017;26(6):743-752. doi:10.1002/hec.3353 27174417

[12] Apantaku GO, McDonald PJ, Aguiar M, et al. Clinician preferences for neurotechnologies in pediatric drug-resistant epilepsy: a discrete choice experiment. Epilepsia. 2022;63(9):2338-2349. doi:10.1111/epi.17328 35699675 PMC9796345

[13] Blasi L, Bordonaro R, Serretta V, Piazza D, Firenze A, Gebbia V. Virtual clinical and precision medicine tumor boards-cloud-based platform-mediated implementation of multidisciplinary reviews among oncology centers in the COVID-19 era: protocol for an observational study. JMIR Res Protoc. 2021;10(9):e26220. doi:10.2196/26220 34387553 PMC8437400

[14] Dharmarajan H, Anderson JL, Kim S, et al. Transition to a virtual multidisciplinary tumor board during the COVID-19 pandemic: University of Pittsburgh experience. Head Neck. 2020;42(6):1310-1316. doi:10.1002/hed.26195 32329958 PMC7264555

[15] Elkaddoum R, Kourie HR, Kassis NE, et al. Treating cancer patients in times of COVID-19 pandemic: a virtual women cancers multidisciplinary meeting experience. Bull Cancer (Paris). 2020;107(7-8):738-740. doi:10.1016/j.bulcan.2020.05.007 32674933 PMC7305862

[16] Stevenson MM, Irwin T, Lowry T, et al. Development of a virtual multidisciplinary lung cancer tumor board in a community setting. J Oncol Pract. 2013;9(3):e77-e80. doi:10.1200/JOP.2013.000882 23942505 PMC3651575

[17] Munro AJ, Swartzman S. What is a virtual multidisciplinary team (vMDT)? Br J Cancer. 2013;108(12):2433-2441. doi:10.1038/bjc.2013.231 23756866 PMC3694234

